# All-Arthroscopic versus Mini-Open Repair of Small to Large Sized Rotator Cuff Tears: A Meta-Analysis of Clinical Outcomes

**DOI:** 10.1371/journal.pone.0094421

**Published:** 2014-04-11

**Authors:** Liancheng Shan, Dong Fu, Kai Chen, Zhengdong Cai, Guodong Li

**Affiliations:** 1 Postdoctoral Research Station of Biomedical Engineering, School of Life Science and Technology, Tongji University, Shanghai, China; 2 Department of Orthopedics, Tenth People’s Hospital of Tongji University, Shanghai, China; University of Michigan, United States of America

## Abstract

**Purpose:**

The purpose of this study was to compare clinical outcomes of patients with full-thickness small to large sized tears undergoing all-arthroscopic versus mini-open rotator cuff repair.

**Method:**

A literature search for electronic databases and references for eligible studies was conducted through Medline, Embase and Cochrane library between 1969 and 2013.

**Results:**

A total of 12 comparative studies (n = 770 patients) were included. Pooled results showed: there were no differences in function outcome, pain scores, retear rate or the incidence of adhesive capsulitis between all arthroscopic and mini-open repair groups.

**Conclusions:**

There were no differences in outcomes between the arthroscopic and mini-open rotator cuff repair techniques, they should be considered alternative treatment options.

**Level of Evidence:**

Level IV, Meta analysis.

## Introduction

Repair of the rotator cuff was first described nearly a century ago by Codman [1]. The most frequently used methods for rotator cuff repair include the open, mini-open, and arthroscopic techniques. Currently, there is a growing trend toward a more minimally invasive approach facilitated by advances in the use of arthroscopy [2]–[5]. Both the mini-open and all-arthroscopic techniques maintain the integrity of the deltoid origin with minimal incision. The arthroscopic approach offers several advantages, including a smaller incision, easy access to the glenohumeral joint for treatment of intra-articular pathology, less soft tissue dissection, and less potential harm to the deltoid. However, a purely arthroscopic rotator cuff repair requires advanced arthroscopic skills. One of the main advantages of the mini-open rotator cuff repair is deltoid preservation thereby eliminating the risk of postoperative deltoid dehiscence. Several systematic reviews [2]–[5], including one meta-analysis [5] have demonstrated that no significant differences existed in mid- and long-term clinical outcomes between arthroscopic and mini-open repair. However, all the five studies included in that meta-analysis by Morse published in 2008 were not randomized clinical trials (RCTs) and the follow-up was retrospective in nature. This meta-analysis involved 12 studies including 3 RCTs published in 2011, 2012, 2013 respectively comparing the results of both methods on the treatment for rotator cuff tears aimed at investigating whether there were any clinical and radiographic differences between these two methods.

## Materials and Methods

### Identification and Selection of Studies

We carried out a literature search using Medline, Embase and Cochrane databases to identify all articles published between 1969 and 2013 that evaluated the outcomes of patients undertaking either AA (all atthroscopic repair) or MO (mini open repair). The language of the publications was limited in English. Comparative reports relating to both AA and MO were included. The following Medical Subject Headings (MeSH) and terms were used in searching: “rotator cuff tear”, “supraspinatus tendon”, “arthroscopic”, “arthroscopy”, “mini open” and “clinical trials”. The reference lists of each comparative study and previous reviews were manually examined to find additional relevant studies. We also contacted each author of the included studies to identify some more details of the clinical outcomes and further studies on the same topic by e-mail. To minimize any possible selection bias, criteria for inclusion were as follows: English reported papers; a clinical trial comparing AA and MO with a clear description of at least one of the indexes analyzed in this study. Two reviewers independently assessed each of the studies for eligibility for inclusion. Firstly, the title and the abstract were judged by either reviewer and then if it was potentially eligible, the full article would be examined. All disagreements were resolved and a final consensus was reached.

### Data Extraction

Data were extracted independently by two authors subsequently after all the eligible studies were recruited. All pertinent information regarding participants and clinical outcomes were recorded. Participants’ information included the number of patients, age, gender (the rate of males in all participants). The principal outcomes of interest included details of operative time, postoperative functional outcomes (ASES, American Shoulder and Elbow Surgeons; UCLA, University of California at Los Angeles; Constant-Murley score), range of motion, pain score as well as reported complications (retear rate, adhesive capsulitis).

### Study Quality

Based on the Cochrane Bone, Joint and Muscle Trauma Group [6], assessment of the methodological quality of each included study was made by the 2 reviewers who were blinded with respect to the journal, the authors and the source institution. Any controversy was cross-checked and resolved by a third author to reach a final consensus.

### Statistical Analysis

Study-specific RR (Ratio Risks) and associated 95% CI (confidence intervals) accounting for discontinuous variables within the study were pooled using a random-effects model, which considered both within-study and between-study variation. Standardized mean difference (SMD) was used for continuous variables for which a fixed effect model was used initially. Heterogeneity is expressed as P and I^2^. This value of I^2^ ranges from 0% (complete consistency) to 100% (complete inconsistency). If the P value of heterogeneity test was <0.1 or I^2^>50% [7], the random-effect model replaced the fixed modality. Sensitivity analysis was performed to evaluate the stability of the results if necessary. Subgroup analysis was conducted to get some more concrete conclusions if the data was present. Forest plots were used to graphically present the results of individual studies and the respective pooled estimate of effect size. All statistical analyses were performed with Review Manager (version 5.0.0 for Windows, The Cochrane Collaboration, The Nordic Cochrane Centre, Copenhagen, 2008).

## Results

### Study Characteristics

**Figure 1:**
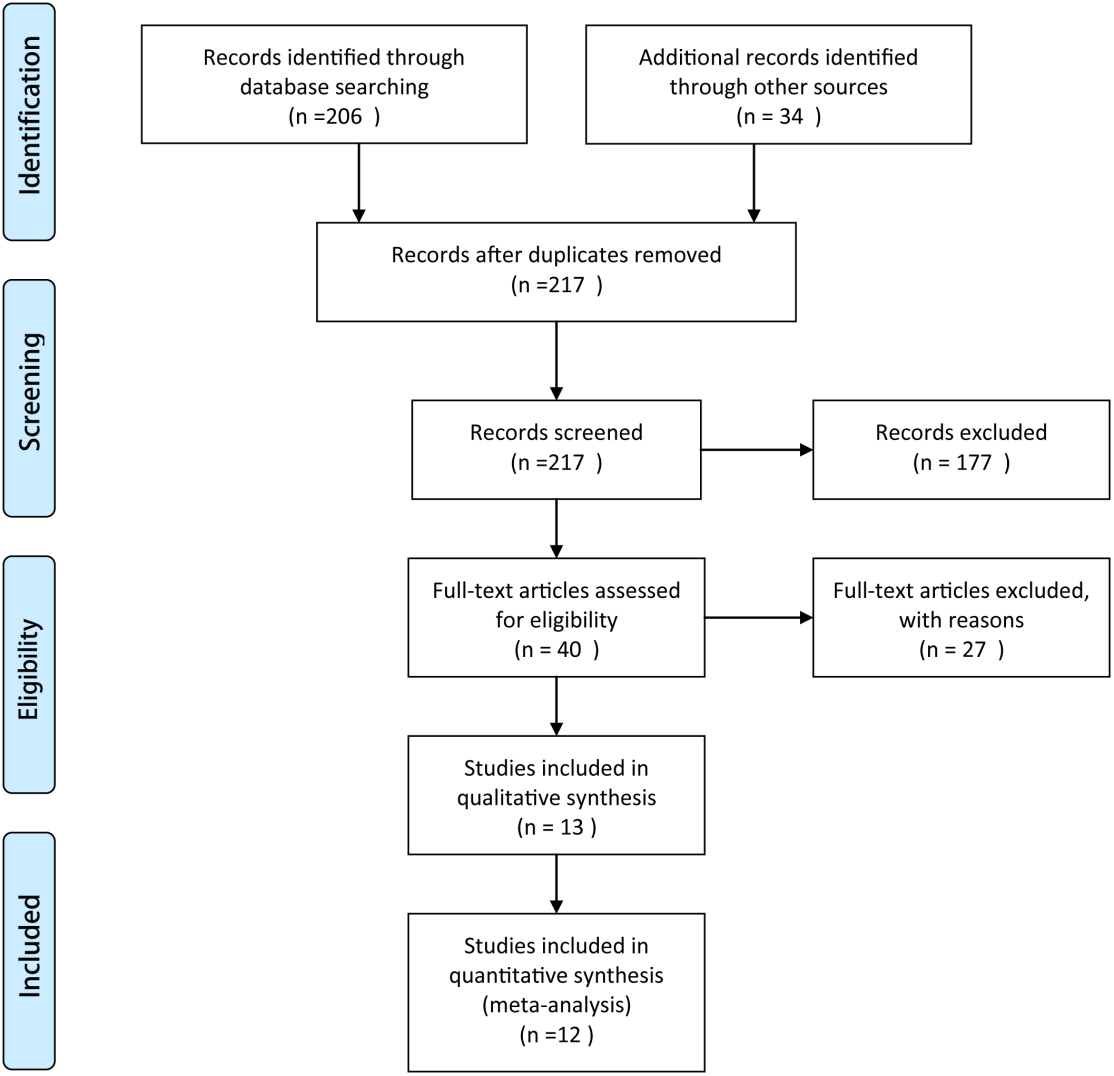
Flow chart of the studies recruited in this meta-analysis.

A flow chart of the studies recruited in our review was shown in Figure 1 (Fig. 1). We identified 240 potential citations (162 from Pubmed; 26 from Embase; 18 from the Cochrane Randomized Trials Databases; and 34 from relevant journals) aiming at comparing AA and MO in the treatment of rotator cuff tears. After reading the articles, as well as communicating with the first author to get additional studies or data, 13 of the 240 citations were selected for application. There was one study belonging to the same institution with different follow-up and only the recent study was selected. As a result, 12 articles [8]–[19] including a total of 770 patients (422 in the AA group and 348 in the MO group) were identified in the final analysis. Demographic information for members of each group was provided in Table 1. The 12 studies on the two different treatment choices for rotator cuff tears were published between 2003 and 2013 (Table 1). Preoperative patient characteristics did not show any significant difference between these two groups with respect to the number of patients, gender and age.

**Table 1:** Demographic information for members of each group.

author	year	Type of study	Group	No	F/M	Age	Follow-up	OT	UCLA S	ASES S	Consant S	VAS S	Flextion	Rotation	Abduction	Retear	AC	Evidence
Zwaal et al	2013	RCT	ASR	47	18/29	57.2y	56w	73.5±17.6	NG	NG	73.5±17.6	NG	170±2.6	80±2.0	NG	8	5	III
			MOP	48	20/28	57.8y	56w	53.8±13.7			53.8±13.7		159±4.3	72±2.9		6	6	
Cho et al	2012	RCT	ASR	30	13/17	55.5y	6m	57.67±11.04	NG	NG	NG	1.07±1.08	159.50±8.74	52.83±7.39	NG	NG	5	III
			MOP	30	13/17	56.2y	6m	61.0±14.65				1.13±1.07	157.67±15.8	53.0±11.11			4	
Kasten et al	2011	RCT	ASR	17	8/9	60.1y	6m	NG	NG	81±20	NG	2.0±1.5	160±15	86±10	NG	3	NG	III
			MOP	17	5/12	60.1y	6m			86.9±16.3		1.2±0.8	164±7	75±18		3		
Stone et al	2009	prospective	ASR	92	48/44	57y	24m	NG	NG	NC	76.58	NG	NG	NG	NG	NG	NG	II
			MOP	31	15/16	62y	24m				69.14							
Kose et al	2008	retrospective	ASR	25	18/7	55y	31.20m	NG	29.76±4.5	NG	83.56±11.45	NG	NG	NG	NG	NG	NG	II
			MOP	25	21/4	62y	21.56m		28.8±3.42		79.56±13.64							
Liem et al	2007	retrospective	ASR	19	3/16	61.9y	25.0m	NG	NG	NG	83.9	NC	176	59	173	NG	4	II
			MOP	19	3/16	62.1y	17.6m				83.7		175	56	175		2	
Verma et al	2006	retrospective	ASR	38	16/22	59.4y	24m	NG	NG	94.6±8.9	NG	0.7±1.2	170.5±6.9	68.2±16.7	169.6±7.5	9	1	II
			MOP	33	10/23	60.7y	24m			95.1±5.3		0.4±1.0	169.4±6.9	70.2±14.	168.9±8.4	9	0	
Andreas et al	2005	retrospective	ASR	26	10/16	56y	19m	NG	NG	86(43–100)	NG	NG	NG	NG	NG	NG	16	II
			MOP	28	12/16	57y	33m			89(56–100)							15	
Warner et al	2005	retrospective	ASR	9	4/5	53y	44m	NG	NG	NG	NG	NG	160 (130–170)	50 (30–60)	NG	NG	NG	II
			MOP	12	4/8	55y	44m						155 (110–170)	50 (25–60)				
Youm et al	2005	retrospective	ASR	42	NG	60	37.6m	NG	33.2±2.5	91.1±15.4	NG	NG	NG	NG	NG	NG	NG	II
			MOP	42		59	37.6m		32.3±3.3	90.2±14.8								
Kim et al	2003	retrospective	ASR	42	15/27	55	39m(24–72)	NG	NG	95±7.2	NG	0.7±1.1	NG	NG	NG	0	0	II
			MOP	34	22/12		39m(24–72)			95±7.3		1.0±1.5				1	4	
Severud et al	2003	retrospective	ASR	35	NG	NG	44.6m	NG	32.6	91.7	NG	NG	NG	NG	NG	NG	NG	II
			MOP	29			44.6m		31.4	90								

Abbreviations: Year = year of publication; OT = operative time; RCT = randomized clinical trials; ASR = arthroscopic repair; MOP = mini open repair; UCLA S = University of California at Los Angeles Score; ASES S =  American Shoulder and Elbow Surgeons index Score; Constant S = Constant-Murley score; VAS = visual analog scale pain score;AC = adhesive capsulitis; NG = not given.

### Outcome Measurements

The results of RR/SMD and 95% CI for each comparison were shown in Table 2. Because of lacking consistency in the way the outcomes were acquired (e.g. the time points when the measurements were conducted differed among the studies), almost all the results were obtained directly from the pooling of data without stratifying by different periods. Moreover, not all studies provided necessary data, so we only compared with the limited articles which might contribute bias to our final results.

**Table 2:** Outcome measures in the meta-analysis of comparisons between all arthrosopic and mini-open cuff tear repair.

Outcome	Risk ratio (95% CI)	P	STD (95% CI)	Heterogeneity	*I* ^2^%	Number of patients	Nubmer of studies
UCLA score		0.11	0.28 (−0.06, 0.62)	0.85	0	134	2
AESE score		0.74	−0.04(−0.28, 0.20)	0.83	0	270	4
Consant score		0.12	0.91 (−0.24, 2.05)	0.002	90	145	2
VAS score		0.59	−1.55(−2.01, 0.53)	<0.00001	91	336	5
Forward flextion		0.29	0.76 (−0.66, 2.17)	<0.00001	96	260	4
External rotation		0.21	0.94 (−0.53, 2.40)	<0.00001	96	260	4
Retear	0.99(0.57, 1.73)	0.97		0.76	0	280	4
Adhesive capsulitis	1.12 (0.76, 1.64)	0.57		0.52	0	394	6

Abbreviation: STD = Std Mean Difference; VAS = visual analog scale pain score; ASES =  American Shoulder and Elbow Surgeons; UCLA = University of California at Los Angeles.

### Functional Results (UCLA Score, AESE Score, Constant Score)

7 studies [8]–[10], [12], [14], [17], [18] using different score systems were involved when comparing the function score between two groups. There was no difference in the function outcome scores between the two groups. Even though at different periods of follow-up, patients got the similar function score measured as UCLA (STD = 0.28 95% CI −0.06, 0.62), AESE (STD  = −0.04 95% CI −0.28, 0.20) and Constant (STD = 0.91 95% CI −0.24, 2.05). Several studies in which only mean value was provided were not included. However, authors in these studies gave homologous results as our outcome.

### Range of Motion (Forward Flextion, External Rotation)

**Figure 2:**
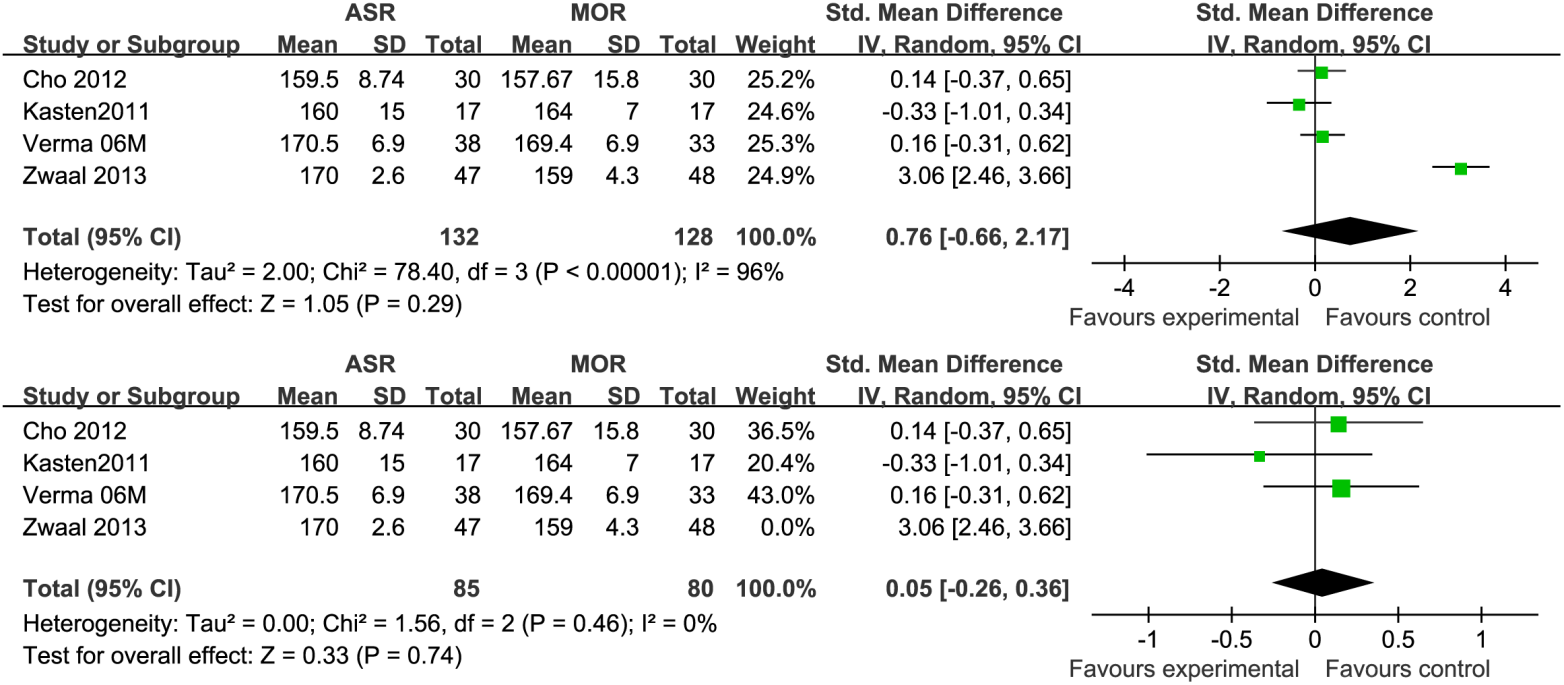
Forest plot showing the SMD (Standardized mean difference) and 95% CI for Forward flextion after surgery with and without the study by Zwaal^13^ during a sensitivity analysis. The heterogeneity decreased significantly when the study by Zwaal et al was excluded.

**Figure 3:**
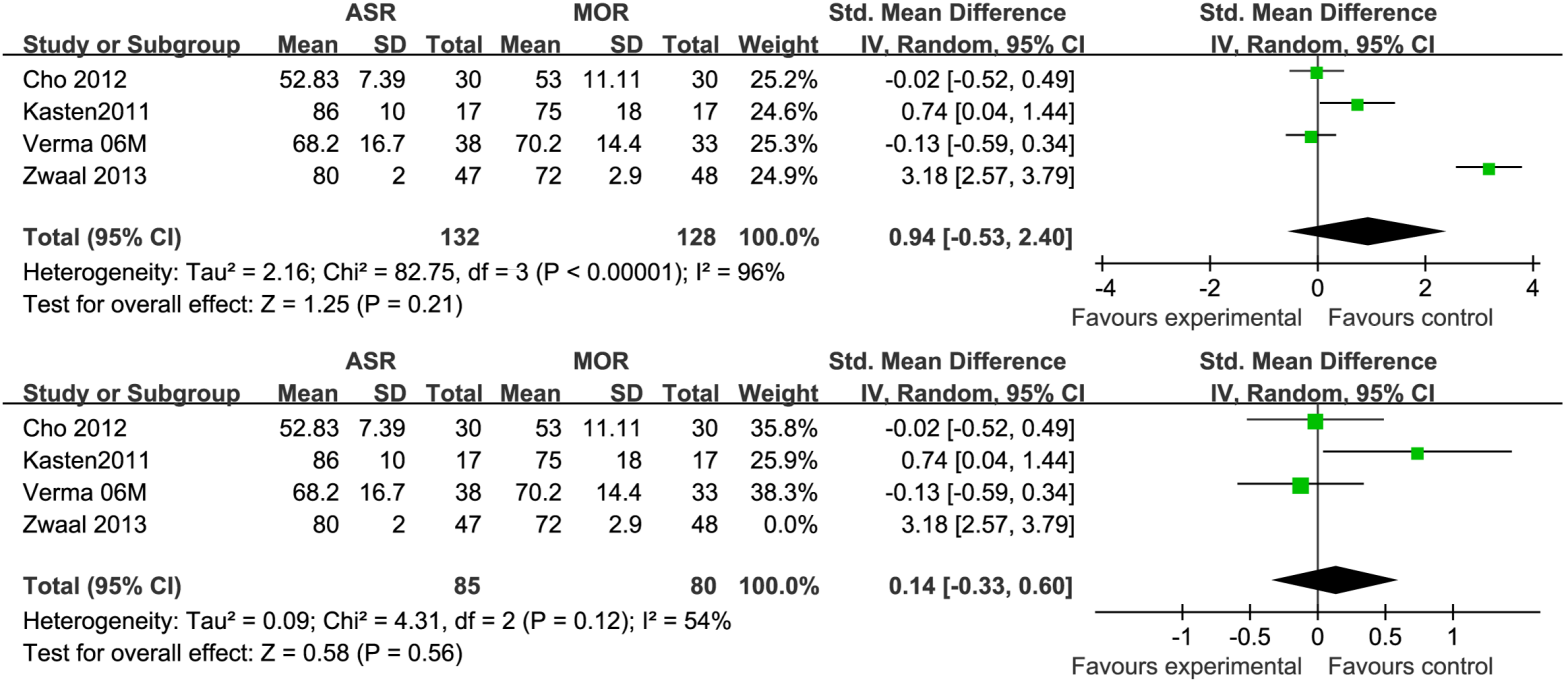
Forest plot showing the SMD (Standardized mean difference) and 95% CI for External rotation after surgery with and without the study by Zwaal^13^ during a sensitivity analysis. The heterogeneity decreased significantly when the study by Zwaal et al was excluded.

Based on the available data, only 4 studies [8]–[10], [14] provided the information about postoperative range of motion (Forward flextion, External rotation) with 260 patients. Among these 4 studies, only one examined the degree of abduction as mean and standard deviation. No statistical difference was observed in either forward flextion (STD = 0.76 95% CI −0.66, 2.17) or external rotation (STD = 0.94 95% CI −0.53, 2.40). During analysis, an obvious heterogeneity (p<0.0001) of the combined data was found. Each of the 4 studies was excluded respectively to do a sensitivity analysis. The result showed that the heterogeneity decreased only when the study published in 2013 by Zwaal et al [8] excluded (Fig. 2, 3). Zwaal reported a significant greater range of motion in both flextion and external rotation in AA group. We tried to find the potential difference between this one and the other three studies and found that patients with simultaneous lesions of the shoulder were excluded by Zwaal. Another possible reason was that all arthroscopic repair would provide a more rapid rehabilitation and better function with advanced techniques and skills.

### VAS Score

A total of 5 studies [8]–[10], [14], [18] including 336 patients were included for analysis of pain score (visual analog scale pain score was chosen as the Uniform standards). Two studies measured this value at 6 month follow-up and the other three gave the data at 12m, 24m, 39m respectively. The 95% CI ranged from −0.21 to 0.53 and the value of Std Mean Difference was 0.59. No significant difference was seen in regards to VAS score. Like comparison of range of motion, an obvious heterogeneity (p<0.0001) occurred in VAS analysis which disappeared while excluding the study by Zwaal et al [8]. The reason might equally own to arthroscopic development.

### Compliation

Seven studies [8]–[10], [13]–[15], [18] and 428 cases were available for the analysis of complications. Even though most previous studies have showed a difference between AA and MO in complications, no significant difference was seen in this meta analysis with respect to postoperative incidence of retear (OR = 0.99, 95% CI 0.57, 1.73) or adhesive capsulitis (OR = 1.12, 95% CI 0.76, 1.64) without any heterogeneity.

## Discussion

Arthroscopy was first used in the treatment of rotator cuff tears as a diagnostic tool, enabling the surgeon to diagnose glenohumeral pathology [20]. Interest in an all-arthroscopic technique has increased in recent years as arthroscopic techniques continue to improve and advance. Mini-open repair represent an attempt to combine the advantages of arthroscopic and open repair. The ability to investigate intra-articular pathology and still repair the tendon with bone tunnels without taking down the deltoid origin has made mini open repair a popular technique. Arthroscopic technique is technically more demanding than the mini-open surgery. The mini-open technique provides direct visualization of the cuff repair and allows the surgeons to place the stitches in an open technique, which is more convenient and familiar for surgeons. The mini-open technique also allows for tension absorbing stitches to be placed in the cases where they are needed. Earlier studies published on all-arthroscopic versus mini-open rotator cuff repair did not find any significant difference between the treatment groups [14]–[17], [19], [21]. In this meta-analysis, we demonstrate similar results, with no differences noted in clinical outcomes between the arthroscopic group and the mini-open group for all scoring scales evaluated. Our analysis is the first high-quality study with the largest number and highest level of articles.

However, some studies [8], [10], [21] have demonstrated that the all-arthroscopic procedure does obtain its treatment effect faster than the mini-open procedure with respect to improvement in function score, VAS pain/impairment scores, and range of motion within 6m postoperatively. None of the other studies reported postoperative pain assessments within such a short time after surgery. This could be attributed to the greater compromise of deltoid muscle tissue in the MO group resulting from increased swelling and detachment of muscle fibers from the acromion [22]. Given that decreased postoperative pain is often associated with a quicker return to work, further study to confirm these results would be helpful in recommending all arthroscopic repair on the basis of decreased postoperative pain. For the 7 studies with a minimum follow-up of 2 years included in this meta-analysis, there were no statistically significant differences in preoperative functional or pain score between groups within each study.

In terms of postoperative adhesive capsulitis, AA offers some advantages over the MO repair. The mini-open approach requires a split in the deltoid fibers extending into the subdeltoid bursa, even though the deltoid origin is preserved. This may lead to subacromial scarring and stiffness [23].Using three to four portal sites instead of a 3to 4 cm incision, arthroscopic technique can potentially reduce the risk of this complication. Because the deltoid origin is preserved during mini open repair, rehabilitation of the shoulder can be less restricted as it is not necessary to wait until the deltoid is healed to the acromion. Currently, with the help of postoperative rehabilitation protocol made by special physical therapist, patients get a quick rehabilitation in both groups and no difference in adhesive capsulitis was detected in this analysis.

Another important issue to patients and their physicians is the recurrent rotator cuff tear. Despite many published concerns regarding long-term integrity of repair [24], [25], our study comparing all-arthroscopic methods to mini-open techniques with mixed populations (small, middle and large tears) showed no difference in incidence of recurrent tears. While exploring the incidence of retear after arthroscopic rotator cuff repair alone, some studies have shown excellent function outcome [26], [27]. The correlation of functional results with integrity of the cuff is worth attention. Liu and Baker [28] presented an interesting result after a minimum follow-up of 2 years. Patients with larger tears had a higher incidence of persistent defects, but there was no significant difference in function outcome between patients with and without defects. Therefore, they got a conclusion that function outcome was not determined by the integrity of the cuff. In a short-term follow-up, Liem et al [13], have correlated the clinical outcome of arthroscopic rotator cuff repairs with magnetic resonance imaging evidence of repair integrity and demonstrated improvements in clinical outcome, regardless of repair integrity. An interesting finding from Verma [14] was that retear rate was higher for the open group and lower for the arthroscopic group for tears smaller than 3 cm, for tears larger than 3 cm, retear rate was lower for the open group and higher for the arthroscopic group. However, this difference was not statistically significant. Although we did not compare outcomes of rotator cuff repair based on tear size in the present study, some factors influencing the high rotator cuff retear rate can’t be ignored including tear size, preoperative duration of symptoms, degeneration of cuff, fixation technique or hardware used.

Arthroscopic repair of the rotator cuff is technically demanding and requires a long learning curve for a surgeon to become specialist in this domain [13], [29]. The mini-open surgical repair technique has a various repair choices from bone tunnels to implantable suture anchors, while only the implantable suture anchor devices can be used for all-arthroscopic surgical technique which may result in the all-arthroscopic rotator cuff repair costing more time [30]. Conversion to a mini-open or open approach is easily done if needed. Because of the technical demands of arthroscopic repair, many researchers consider one of the most obvious advantages of the mini open repair procedure is that it consumes less operative time. 2 [8], [10] of the 12 included studies gave the data about operative time. One was published in 2013 [8], the mean time of surgery were 73.5 min and 53.8 min for AA and MO repair respectively with a dominance of reduced 20 min for MO group. Another study published in 2012 [10], conversely a shorter operative duration was obtained in AA group (57.67 min versus 61.00 min). Neither of these two studies showed any significant difference about surgery time. With recent innovations and technologic advances of general arthroscopic instruments and rotator cuff repair-specific appliances, arthroscopic repair does achieve a good clinical result with shorted operative time.

## Limitation

Some limitations must be recognized in this meta-analysis. First of all, in spite of this fact that we have included all the eligible comparative studies, the number of patients is relatively small. Second, some parameters (eg. Operative time, the degree of abduction, total cost) were not analyzed as a result of insufficient data. Furthermore, there were many variations of the 12 included studies. For instance, specific inclusion and exclusion criteria varied, as some studies included patients who had undergone other procedures for additional intra-articular pathology during surgery, whereas others did not. Some use bone tunnels and others use suture anchors for osseous fixation in mini open group. Future studies should address this issue.

## Conclusion

This Meta analysis demonstrated that arthroscopic and mini-open repair produce equivalent clinical outcomes in the middle to long postoperative period. Overall, both arthroscopic and mini open techniques are effective and viable options for surgeons with advanced arthroscopic skills.

## Supporting Information

Checklist S1 PRISMA Checklist.
(DOC)
